# Exposure to anti- and pro-smoking messages among adults in China: Results from the Global Adult Tobacco Survey, 2018

**DOI:** 10.1371/journal.pone.0304028

**Published:** 2024-06-13

**Authors:** Di Pei, Lucy Popova, Pranesh Chowdhury, Jing Shi, Gibril Njie

**Affiliations:** 1 School of Public Health, Georgia State University, Atlanta, Georgia, United States of America; 2 Office on Smoking and Health, National Center for Chronic Disease Prevention and Health Promotion, Centers for Disease Control and Prevention (CDC), Atlanta, Georgia, United States of America; 3 Noninfectious Disease Programs, CDC Foundation, Atlanta, Georgia, United States of America; University of Pennsylvania, UNITED STATES

## Abstract

**Significance:**

For decades, tobacco advertisements and promotions have been common in mass media and public places in China. In 2015, China amended the Advertising Law to prohibit the distribution of tobacco advertising, while also initiating waves of tobacco control media campaigns. This study investigates the associations between exposure to anti- and pro-smoking messages, smoking status, and people’s smoking-related beliefs and willingness to support tobacco control policies.

**Methods:**

A secondary data analysis was performed with the 2018 Global Adult Tobacco Survey of 19,376 adults aged ≥15 years in China. Anti- and pro-smoking message exposures were measured as the sum of sources (media or places) where respondents have seen the messages. Multivariable logistic regression analyses were conducted to examine the relationships among smoking status, message exposure, and the outcome variables (health harm beliefs, support for increasing tax on cigarettes, support for using part of the increased tax on tobacco control) controlling for smoking status and demographic differences.

**Results:**

Overall, 63.3% of the respondents reported being exposed to anti-smoking messages from at least 1 source, while 18.1% were exposed to pro-smoking messages from at least 1 source. Adults who currently, formerly, and never smoked differed in their beliefs about smoking and willingness to support tobacco control policies. Greater reported exposure to anti-smoking messages was positively associated with belief that smoking is harmful, support for increased cigarette tax, and support for using increased tax revenue for tobacco control measures. Meanwhile, greater reported exposure to pro-smoking messages was negatively related to willingness to support cigarette tax increases.

**Conclusions:**

While national and local tobacco control campaigns in China have reached a large proportion of the adult population, there is still room for improvement. China might consider expanding anti-tobacco campaigns, as reported exposure to these messages is associated with increased public awareness of the health hazards of smoking and support for increasing cigarette taxes.

## Introduction

Tobacco use is one of the leading causes of premature death, annually causing over 8 million deaths globally [1]. In China, in 2018, an estimated 307.6 million Chinese adults smoked tobacco daily or occasionally, which accounts for 26.6% of its population [2]. In an effort to effectively address the public health challenges caused by tobacco use, the World Health Organization Framework Convention on Tobacco Control (WHO FCTC) was created. This public health treaty helps countries reduce tobacco use and exposure to tobacco smoke through evidence-based practices and recommendations. China signed the treaty in 2003 and it was ratified in 2005, showing the country’s commitment to implement the provisions outlined in the WHO FCTC [3]. Article 13 of the WHO FCTC mandates a complete prohibition on tobacco advertising, promotion, and sponsorships (TAPS) [4]. To facilitate the implementation and enforcement of the WHO FCTC by the parties, WHO developed article implementation guidelines and created the MPOWER policy package to guide efforts to reverse the tobacco epidemic [5].

The MPOWER policy package is a set of six cost-effective, evidence-based strategies for reducing tobacco demand [6]. Article 13 of the WHO FCTC and the MPOWER strategies both recognize the importance of a comprehensive ban of TAPS [4, 6]. Pro-smoking messages are popular marketing strategies that tobacco companies use to target potential and existing customers. Previous studies have documented that pro-smoking messages not only promote smoking initiation, they also reduce intention to quit among people who currently smoke and encourage relapse among people who formerly smoked [7, 8]. In China, pro-smoking messages have been distributed in mass media and public places with high volume and wide coverage for decades [9]. As part of the tobacco control efforts, in 2015, China amended he Advertising Law to prohibit the distribution of tobacco advertising in mass media and public places [10]. However, because “public places” was not clearly defined in the law, and there was little regulation of sponsorships and retailer promotions, tobacco companies continued promoting tobacco products through avenues not specified in the ban [11, 12]. Recent studies have revealed that Chinese adolescents are still commonly exposed to tobacco ads at retail stores and kiosks near their schools, subway and bus stations, and shopping malls [13, 14].

In addition to the policy of prohibiting TAPS, the MPOWER measures suggest other tobacco control strategies, such as warning the public about the dangers of tobacco use and increasing taxes on tobacco. Mass communication campaigns are a common approach to increasing public awareness of the dangers of tobacco use. Studies in different countries suggest that exposure to anti-smoking messages increases the perceived harm of smoking and discourages tobacco use [15–17]. Moreover, exposure to effective anti-smoking messages is associated with increased support for tobacco control policies, including higher cigarette taxes [18–20]. In China, since 2014, evaluations of national and city-based anti-smoking campaigns suggest that these efforts have informed the public about the harms of smoking and encouraged cessation among people who smoke [21–24].

Implementing a TAPS ban and initiating waves of anti-smoking campaigns are expected to change the Chinese public’s beliefs about the health hazards of smoking and their willingness to support tobacco control policies. Existing studies that examined the relationship between smoking-related message exposure and people’s attitudes and behavioral intentions were mostly conducted in selected cities or regions of China, while nationally representative studies reported only overall smoking-related message exposure rates by channel [5, 25]. Our study aims to fill the gap by 1) analyzing data from the most recent 2018 Global Adult Tobacco Survey (GATS) in China and 2) examine the relationships among smoking status, reported exposure to anti- and pro-smoking messages, and adults’ health harm beliefs and support for tobacco control policies.

## Method

### Study sample

We conducted secondary data analysis using the 2018 GATS from China (GATS China 2018), the most recent data available. GATS is a nationally representative, cross-sectional household survey of adults aged ≥15 years to monitor tobacco use and track key tobacco control measures. GATS uses a multi-stage, stratified, probability sampling process, whereby households were randomly selected within each statistical region in the country, and one adult aged ≥15 years was randomly selected from each household. The survey used standardized protocols and data collection instruments; selected survey respondents were interviewed in person by a trained interviewer [26]. Additional information about the GATS methodology is available elsewhere [27, 28]. The overall household response rate for GATS China 2018 was 92.7%. A total of 19,376 interviews were completed with an overall response rate of 98.7%. Characteristics of the sample are presented in Table 1. Data collection for GATS China 2018 was approved by the Ethical Committee of the Chinese Center for Disease Control and Prevention. Interviewers obtained verbal consent from all participants. This study was not subject to ethical review since we conducted a secondary analysis of publicly available data.

**Table 1:** Prevalence of exposure to anti-smoking messages and pro-smoking messages among Chinese adults, Global Adult Tobacco Survey, China, 2018.

	Study population^a^	Anti-smoking messages exposure			Pro-smoking messages exposure		
	Number of adults (sample distribution %)	Reported exposure in the past 30 days, %	95% CI	p-value	Reported exposure in the past 30 days, %	95% CI	p-value
**Overall**	19,376 (100.0)	63.3	60.9–65.6	< .001*	18.1	16.4–19.8	< .001*
**Smoking status**							
Current tobacco smoking	4,959 (26.6)	64.0	61.1–66.8	0.05	25.5	22.1–28.8	< .001*
Former tobacco smoking	1,553 (6.7)	66.9	63.3–70.5		16.7	13.1–20.3	
Never tobacco smoking	12,863 (66.7)	62.6	60.1–65.1		15.3	13.8–16.9	
**Gender**							
Men	9,109 (50.6)	66.1	63.6–68.6	< .001*	22.9	20.4–25.4	< .001*
Women	10,267 (49.4)	60.4	57.7–63.0		13.2	11.8–14.6	
**Age (years)**							
15–24	930 (13.9)	75.8	71.4–80.2	< .001*	28.5	23.8–33.3	< .001*
25–44	5,128 (37.8)	69.9	67.3–72.6		20.7	18.5–22.9	
45–64	8,652 (34.7)	56.8	54.2–59.4		14.2	12.4–15.9	
≥65	4,666 (13.7)	48.4	45.5–51.4		10.3	8.6–12.1	
**Residence**							
Urban	11,023 (59.9)	69.8	67.1–72.5	< .001*	19.0	17.0–21.1	0.2
Rural	8,353 (40.1)	53.5	49.5–57.5		16.7	13.9–19.6	
**Education level**							
Elementary school & below	7,542 (28.1)	42.4	39.7–45.1	< .001*	12	10.3–13.7	< .001*
Middle school	6,010 (33.9)	66.4	63.5–69.4		19.2	16.7–21.7	
High school graduate	3,040 (19.1)	72.5	69.5–75.5		22.2	19.1–25.4	
Junior college & above	2,762 (18.9)	79.4	76.5–82.3		20.9	18.2–23.6	

CI, confidence interval.

Anti- and pro-smoking message exposure are categorical variables.

Weighted data are presented unless otherwise specified in the table.

^a^Data reported in this column are unweighted.

* Statistically significant (p < 0.01 based on Rao-Scott chi-squared test).

### Measures

#### Anti-smoking message exposure

The following question in the GATS China 2018 questionnaire was used to measure exposure to anti-smoking messages: “Over the past 30 days, have you seen messages about the harms of smoking or messages to encourage cessation in the following media/places?” Answers included newspapers or magazines, TV, radio, billboards, bulletin boards, posters or promotional materials, internet, and other places. Each source selected by the respondents was coded as 1. A composite score was calculated as the sum of sources noted by the respondent. A categorical “any anti-smoking message exposure” variable was also created to report the overall prevalence of any anti-smoking message exposure. Respondents who were exposed to anti-smoking messages from at least 1 source were coded as 1; those who had no exposure were coded as 0.

#### Pro-smoking message exposure

The following questions were selected as measurements of exposure to pro-smoking messages:

1) “Over the past 30 days, have you seen any advertisement or signs promoting cigarettes in the following places?” Answers included newspapers or magazines, TV, radio, billboard, posters or promotional materials, internet, cinema, public transportation or platform, wall advertisement in public places, and other places.

2) “Over the past 30 days, have you seen any sports activities or events related to cigarette brands or cigarette business?” Answers included yes, no, and don’t know.

3) “Over the past 30 days, have you seen any campaigns or field activities related to cigarette brands or cigarette business in the community?” Answers included yes, no, and don’t know.

4) “In the last 30 days, have you noticed any of the following types of cigarette promotions?” Answers included free samples of cigarettes, cigarettes at sale prices, coupons, free gifts or special discount offers, clothing or other items with a cigarette brand name or logo, promotions in the mail, and single sales.

Each outlet selected by the respondents in questions 1 and 4 was coded as 1. Choosing “yes” in questions 2 and 3 was also coded as 1. A composite score was calculated by summing the scores of the recoded items, representing the amount of exposure to pro-smoking messages. Similar to the anti-smoking message exposure variable, a categorical “any pro-smoking message exposure” variable was created, with 1 representing “any exposure” and 0 representing “no exposure.”

**Health harm beliefs**Respondents were asked: “As far as you know, does smoking cause severe diseases?” Answers included yes, no, and don’t know.

#### Support for increasing tax on cigarettes

Respondents were asked: “Do you support raising tax on cigarettes (raising cigarette retail price)?” Answers included yes, no, and don’t know.

#### Support for using part of the increased tax on tobacco control

Respondents were asked: “If cigarette tax is increased, do you agree that part of the increased tax revenue can be used for tobacco control (e.g., for cessation services and warning the public of tobacco harms)?” Answers included yes, no, and don’t know.

The present study also included some sociodemographic variables as covariates: sex (man/woman), age (15–24, 25–44, 45–64, 65 or older), residence (urban/rural), and education (elementary school and below, middle school, high school graduate, junior college and above). Tobacco smoking status included current smoking (currently smoke tobacco daily or nondaily), former smoking (currently do not smoke but smoked daily or nondaily before), and never smoking (currently do not smoke and did not smoke before).

### Data analysis

Sociodemographic characteristics and tobacco smoking status were reported with weighted prevalence estimates and 95% confidence intervals (CIs). Chi-square tests were used to compare exposure to smoking-related messages among different demographic and smoking status categories. A p-value less than 0.01 was considered statistically significant. Multivariable logistic regression analyses were conducted to examine the relationships among smoking status, reported message exposure, and the outcome variables (health harm beliefs, support for raising cigarette taxes, and support for using increased tax revenue on tobacco control)—after controlling for covariates. Unadjusted odds ratios (UORs) and adjusted odds ratios (AORs) with 95% CIs were reported. The “don’t know” responses were treated as missing values in the analyses. All analyses were performed using SAS version 9.4 (SAS Institute Inc, Cary, NC), and accounted for the complex survey design.

## Results

Overall, 63.3% of Chinese adults reported seeing anti-smoking messages, and 18.1% reported being exposed to pro-smoking messages in the past 30 days (Table 1). Reported exposure to both anti-smoking messages and pro-smoking messages was significantly different across current smoking status and some demographic groups: gender, age, and education. Specifically, prevalence of anti-smoking message exposure was 64.0%, 66.9%, and 62.6% among adults who currently, formerly, and never smoked, respectively (p = 0.05). Prevalence of pro-smoking message exposure in the past 30 days was significantly different (p<0.001) by smoking status: 25.5%, 16.7%, and 15.3% of adults who currently, formerly, and never smoked, respectively.

Prevalence of anti- and pro-smoking message exposure was significantly different for men and women (p<0.001). More Chinese men reported being exposed to anti-smoking (66.1%; 95% CI, 63.6%–68.6%) and pro-smoking messages (22.9%; 95% CI, 20.4%–25.4%) compared to women (60.4%; 95% CI, 57.7%–63.0%; 13.2%; 95% CI, 11.8%–14.6%). Furthermore, prevalence of anti- and pro-smoking message exposure was significantly different among different age and educational attainment groups (Table 1). The highest percentage of exposure was reported among young adults aged 15–24 years, with 75.8% of them reported being exposed to anti-smoking messages (95% CI, 71.4%–81.2%) and 28.5% reported being exposed to pro-smoking messages (95% CI, 23.8%–33.3%). Those with a junior college or higher degree had the highest percentage of anti-smoking exposure (79.4%; 95% CI, 76.5%–82.3%), while those with a high school degree had the highest percentage of pro-smoking exposure (22.2%; 95% CI, 19.1%–25.4%).

As shown in Table 2, among Chinese adults, 86.0% (95% CI, 84.6%–87.4%) were aware of the health hazards of smoking. The percentages of adults who believed smoking is harmful to health were significantly different by smoking status: 81.4%, 90.1%, and 87.4% of adults who currently, formerly, and never smoked, respectively. When asked whether they would support increasing tax on cigarettes, 41.8% of Chinese adults responded yes (95% CI, 39.6%–44.0%). Such support for increased cigarette taxes also differed significantly by smoking status: 26.0%, 51.4%, and 47.1% of adults who currently, formerly, and never smoked, respectively. Furthermore, 72.8% of Chinese adults overall supported using part of the increased tax money on tobacco control efforts (95% CI, 70.5%–75.0%), with the percentages 64.6%, 81.0%, and 75.2% among adults who currently, formerly, and never smoked, respectively.

**Table 2:** Prevalence of smoking harm belief, support for increasing tax on cigarettes, and support for using increased tax on tobacco control among Chinese adults, Global Adult Tobacco Survey, China, 2018.

	Smoking harm belief			Supporting increasing tax on cigarettes			Using part of cigarette tax money on tobacco control		
	%	95% CI	p-value	%	95% CI	p-value	%	95% CI	p-value
**Overall**	86.0	84.6–87.4	< .001*	41.8	39.6–44.0	< .001*	72.8	70.5–75.0	< .001*
**Smoking status**									
Current tobacco smoking	81.4	79.5–83.3	< .001*	26.0	24.0–28.0	< .001*	64.6	61.6–67.6	< .001*
Former tobacco smoking	90.1	88.2–92.1		51.4	47.3–55.4		81.0	78.2–83.9	
Never tobacco smoking	87.4	85.9–89.0		47.1	44.4–49.8		75.2	72.8–77.6	
**Gender**									
Men	85.5	84.1–86.9	0.1	39.0	36.8–41.2	< .001*	70.7	68.0–73.4	< .001*
Women	86.5	84.8–88.2		44.6	41.8–47.3		74.9	72.6–77.1	
**Age (years)**									
15–24	93.7	91.3–96.1	< .001*	40.4	35.4–45.3	< .001*	75.3	70.4–80.1	< .001*
25–44	90.7	89.2–92.1		46.0	43.5–48.5		76.9	74.6–79.1	
45–64	82.7	81.0–84.4		39.4	36.9–41.8		71.0	68.4–73.6	
≥65	73.8	70.9–76.6		37.6	34.7–40.5		63.4	60.3–66.5	
**Residence**									
Urban	89.0	87.6–90.4	< .001*	46	43.2–48.7	< .001*	76.5	74.3–78.7	< .001*
Rural	81.6	79.1–84.0		35.5	32.3–38.7		67.2	62.8–71.7	
**Education Level**									
Elementary school & below	74.5	72.1–77.0	< .001*	31.6	28.6–34.6	< .001*	63.2	59.8–66.6	< .001*
Middle school	87.8	86.0–89.5		38.9	36.3–41.4		73.4	70.5–76.4	
High school graduate	92.4	91.1–93.8		45.9	42.7–49.1		76.2	73.1–79.3	
Junior college & above	93.6	92.1–95.0		58.2	54.8–61.5		82.7	80.5–84.9	

CI, confidence interval.

* Statistically significant (p < 0.01 based on Rao-Scott chi-squared test).

Results of multivariable logistic regressions are presented in Table 3. While adjusting for both reported anti- and pro-smoking messages exposure and sociodemographic differences, adults who formerly smoked were more likely to believe smoking is harmful to health (AOR = 2.59; 95% CI, 2.06–3.24), compared with adults who currently smoked. Both adults who formerly smoked (AOR = 3.12; 95% CI, 2.57–3.79) and adults who never smoked (AOR = 2.91; 95% CI, 2.47–3.43) were more likely to support increasing tax on cigarettes, compared to adults who currently smoked (Table 3). Moreover, adults who formerly smoked (AOR = 2.59; 95% CI, 2.12–3.15) were more likely to support using part of the increased tax on tobacco control efforts. Adults who never smoked and those who currently smoked did not differ in their beliefs about the health hazards of smoking and their willingness to support using the increased tax on tobacco control.

**Table 3:** Association of health harm belief, support for increased tax on cigarettes, and support for using tax on tobacco control—by smoking status and message exposure—Global Adult Tobacco Survey, China, 2018.

	Smoking harm belief				Support increasing tax on cigarettes				Support using part of cigarette tax money on tobacco control			
	UOR	95% CI	AOR^1^	95% CI	UOR	95% CI	AOR^1^	95% CI	UOR	95% CI	AOR^1^	95% CI
**Smoking Status**												
Current tobacco smoking	Ref.	-	Ref.	-	Ref.	-	Ref.	-	Ref.	-	Ref.	-
Former tobacco smoking	2.09*	1.68–2.60	2.59*	2.06–3.24	3.00*	2.50–3.61	3.12*	2.57–3.79	2.34*	1.94–2.83	2.59*	2.12–3.15
Never tobacco smoking	1.59	1.37–1.84	1.54	1.26–1.89	2.53*	2.22–2.88	2.91*	2.47–3.43	1.66	1.47–1.87	1.51	1.28–1.78
**Reported exposure to anti-smoking message**	1.50*	1.42–1.59	1.35*	1.28–1.43	1.15*	1.12–1.19	1.10*	1.07–1.13	1.23*	1.18–1.28	1.18*	1.13–1.22
**Reported exposure to pro-smoking message**	1.28*	1.13–1.44	1.05	0.94–1.18	0.98	0.93–1.03	0.93*	0.89–0.98	1.04	0.98–1.11	0.95	0.89–1.02

AOR, adjusted odds ratio; CI, confidence interval; UOR, unadjusted odds ratio.

Note:

^1^ Odds ratio adjusted for demographic variables, smoking status, exposure to anti-smoking and pro-smoking messages. Exposure to anti-smoking message and exposure to pro-smoking message are continuous variables.

- Not applicable.

*Significant at p < 0.01.

When adjusting for both smoking status and demographic differences, reported exposure to anti-smoking messages from more sources was positively associated with belief that smoking is harmful (AOR = 1.35; 95% CI, 1.28–1.43), supporting increased tax (AOR = 1.10; 95% CI, 1.07–1.13), and supporting the use of increased tax on tobacco control efforts (AOR = 1.18; 95% CI, 1.13–1.22). Meanwhile, when adjusting for both smoking status and demographic differences, reported exposure to pro-smoking messages from more sources was negatively associated with supporting increased tax on cigarettes (AOR = 0.93; 95% CI, 0.89–0.98).

## Discussion

Our results show that more than half of Chinese adults (63.3%) have reported seeing anti-smoking messages in mass media and/or public places in the past 30 days, while 18.1% of Chinese adults have reported seeing pro-smoking advertisements or promotions. In the GATS China 2010 survey report, the prevalence of seeing anti-smoking messages in mass media and/or public places in the past 30 days was 46.4% [29]. As a result of the national-level tobacco control efforts since 2010, more Chinese adults have reported being exposed to anti-smoking information in their daily life. However, as reported in a previous study [25], although the prevalence of pro-smoking message exposure showed a slight decrease from 2010 to 2018 (19.6% vs. 18.1%), the difference was not statistically significant. Research suggests that comprehensive TAPS bans have a significant impact on reducing tobacco consumption, while a limited set of TAPS bans would have little or no effects on consumption [30, 31]. Therefore, China might consider enhancing and enforcing its current ban on tobacco advertising and promotions to reduce exposure to pro-smoking messages, thus strengthening its tobacco control regulations and reducing tobacco demand.

Our results also showed that the majority (86%) of Chinese adults believe smoking is harmful: 81.4%, 90.1%, and 87.4% of adults who currently, formerly, and never smoked, respectively. However, as discussed in some studies, although people who smoke are aware of its harms and are motivated to quit, their addiction to nicotine makes it difficult for them to quit [32, 33]. Nicotine from tobacco products maintains addiction by affecting the brain’s neurochemistry and developing physiological dependence among users [34]. As previous anti-smoking campaigns in China have focused primarily on informing the public about the harms of smoking and secondhand smoke exposure [21, 22], future campaigns that continue to educate people about the health hazards could be complemented by adding content to help motivate and navigate cessation. Existing research has suggested that higher self-efficacy in quitting is associated with higher quit intention among cigarette users [35–37]. Specifically, an evaluation of a social media tobacco control campaign in China from 2011 to 2015 suggested that perceived risk and efficacy-related postings were positively associated with audience engagement [38].

Reported exposure to anti-smoking messages was positively associated with the belief that smoking is harmful to health, support for increased tax on cigarettes, and use of the increased tax to fund tobacco control efforts. This finding is consistent with previous research [15–19]. In China, national and local level public campaigns may have the potential to increase public awareness of the health hazards of smoking, as well as to gain public support for tobacco control policies. The national and local level public campaigns may consider continuing those anti-smoking messages, as consistent exposure to anti-smoking messages has the potential to also increase cessation intention and attempts among people who smoke. For example, evaluations of the US-based Tips From Former Smokers (Tips) campaign show that exposure to the Tips messages significantly correlated with quit attempts and sustained quitting on a population level [39, 40].

After adjusting for smoking status and demographic differences, our study found that reported exposure to pro-smoking messages was negatively associated with supporting the use of the increased tax on tobacco control efforts. Prior research on the impact of pro-smoking messages has primarily focused on the relationship between message exposure and smoking behavior [30, 31, 41–43], with limited knowledge about the relationship between exposure to pro-smoking messages and willingness to support tobacco control policies. Our results add to the literature by suggesting that reported exposure to pro-smoking messages from more sources is associated with less willingness to support for the use of cigarette tax revenue on tobacco control efforts, which might indicate a potential health benefit of enhancing the advertising ban in China.

Additionally, we found a positive association between reported exposure to pro-smoking messages and believing smoking is harmful before controlling for covariates. After controlling for smoking status and demographic differences, the relationship between reported exposure to pro-smoking messages and the belief that smoking can be harmful to health was not statistically significant. This finding differs from previous study findings, which provide consistent evidence that exposure to pro-smoking messages is associated with lower perceived harms of smoking as well as higher susceptibility to smoking [7, 44–47]. One possible explanation is that the reported exposure to pro-smoking messages in the present study was measured only as the number of sources where participants have been exposed, but not the content of the pro-smoking messages. Many factors can influence the effectiveness of persuasive messages, including message themes, message features, message quality, and source credibility [48–50]. Future research, especially more comprehensive content analysis of the pro-smoking messages after the advertising ban in China, might further explain this finding.

Our study found significant differences among adults who formerly smoked and currently smoked in their beliefs about the health hazards of smoking and their support for an increased tax on cigarettes, which is consistent with previous research conducted in the US [51]. More specifically, adults who formerly smoked reported higher likelihood of believing smoking is harmful to health, supporting increased tax, and supporting using part of the increased tax on tobacco control efforts compared to adults who currently smoked, controlling for exposure to both anti- and pro-smoking messages. Adults who formerly smoked might have developed stronger perceptions about the harms of smoking and support for tobacco control efforts to motivate themselves to quit and to avoid relapse. As suggested in previous research, successful cessation often involves the use of a variety of psychological and behavioral strategies to increase awareness of the risks, motivate quit attempts, and maintain quit intentions throughout the process [52, 53]. Sharing the strategies and experiences of those who formerly smoked may be helpful for those who currently smoke and have the intention to quit. As suggested by the Tips campaign evaluation studies, exposure to ads and campaigns that feature people who formerly smoked can significantly increase the effectiveness of the campaign message [54]. Future tobacco control campaigns and interventions in China may consider involving people who formerly smoked to share their experiences, which may not only increase intention and efficacy in quitting among those who currently smoked, but also provide social support during the cessation process.

This study has some potential limitations. First, we used cross-sectional survey data from GATS. Therefore, the relationships among the main variables should not be interpreted as causal. Second, the responses were self-reported, so they were subject to potential recall and social desirability bias. Nonetheless, measuring message exposure using self-reported data is a common practice in communication research. Previous research suggests that reported exposure tends to be greater in media markets that receive more message placements [55, 56]. Third, the continuous variables for exposure to anti- and pro-smoking messages were created by summing up the number of sources (media and places) where participants encountered messages. It may not reflect the frequency or dosage of exposure and assumes that all channels are equivalent in their impact on viewers. Last, since the GATS interview instruments are standardized in different countries to ensure international comparability, we were not able to adjust variable measurements. However, as a protocol that has been widely adopted and tested over time, the questions were comprehensive and captured the key attributes associated with tobacco use in the global context.

## Conclusion

Over half of the adults in China reported being exposed to anti-smoking messages, and more anti-smoking message exposure was positively associated with 1) the belief that smoking is harmful and 2) support for tobacco control policies. The national and local level tobacco control campaigns seem effective in increasing public awareness of the health hazards of smoking and enhancing support for increased cigarette taxes in China. Meanwhile, over 18% of Chinese adults reported being exposed to pro-smoking messages in 2018, 3 years after passage of the 2015 Amendment of the Advertising Law. Policies and interventions may consider strengthening the definition of public places to comprehensively prohibit cigarette advertising, event sponsorships, and promotions nationwide and developing effective smoking control campaigns to encourage cessation. In addition to educating people about the hazards of smoking, future campaigns may consider other strategies that could potentially increase quit intention and guide quit attempts, such as promoting efficacy in quitting and featuring former smokers to share experiences and provide social support.
